# Inflammatory Response in Oral Biofilm during Pregnancy: A Systematic Review

**DOI:** 10.3390/nu14224894

**Published:** 2022-11-19

**Authors:** Berit Lieske, Nataliya Makarova, Bettina Jagemann, Carolin Walther, Merle Ebinghaus, Birgit-Christiane Zyriax, Ghazal Aarabi

**Affiliations:** 1 Department of Periodontics, Preventive and Restorative Dentistry, Center for Dental and Oral Medicine, University Medical Center Hamburg-Eppendorf, Martinistraße 52, O58, 20246 Hamburg, Germany; 2 Midwifery Science–Health Care Research and Prevention, Research Group Preventive Medicine and Nutrition, Institute for Health Service Research in Dermatology and Nursing (IVDP), University Medical Center Hamburg-Eppendorf, Martinistraße 52, W38, 20246 Hamburg, Germany

**Keywords:** oral biofilm activity, pregnancy, inflammation, biomarkers and metabolomics, lifestyle and nutrition

## Abstract

Understanding the inflammatory response in oral biofilm during pregnancy and its association with oral and maternal health is essential for identifying biomarker patterns that may serve as markers of pregnancy-related complications. We aimed to conduct a systematic review of the available literature to assess: (1) inflammatory responses in oral biofilm during pregnancy, (2) the association between inflammatory responses in oral biofilm during pregnancy and maternal, oral or systemic conditions, (3) changes in the response of inflammatory biomarkers found in the oral biofilm during different pregnancy stages, and (4) the value of other risk factors such as nutrition and lifestyle. PubMed, Web of Science and Cochrane Library were systematically searched from inception until April 2022. From 5441 records, 39 studies were included for qualitative assessment. The oral biofilm in pregnant women was associated with increased inflammatory biomarkers when compared to non-pregnant women. Levels of inflammatory biomarkers in the oral biofilm were found to be highest in pregnant women with systemic conditions. Increased inflammatory biomarkers in the oral biofilm were also associated with worse oral health outcomes. Given the importance of nutrition and lifestyle for pregnancy and oral health outcomes and the fact that these factors were largely excluded in the included studies, future research should consider a holistic view of the mother during pregnancy to capture physiological, hormonal, immunologic, and metabolic changes in the context of inflammatory responses.

## 1. Introduction

Pregnancy is a unique state of continuous adaptation of the maternal physiology, including changes in women’s metabolism, hormone levels, inflammatory state, nutritional needs, and immune system [1]. These complex changes may also affect the oral cavity by altering the composition of the oral microbiome, which may lead to dysbiosis of the oral biofilm, thereby increasing pregnant women’s susceptibility to oral diseases, such as gingivitis or periodontitis [2]. The oral biofilm is functionally and structurally organized and found naturally in states of health. However, due to hormonal changes and differing metabolic processes occurring during pregnancy, active oral pathogens may easily grow and propagate in the oral cavity [3], thereby breaking down this relationship. As a consequence, disease, stemming from microbial biofilms formed on the tooth surface, may occur [3]. In turn, oral diseases are related to serious local and systemic disorders.

An unbalanced microbial biofilm has been shown to trigger systemic inflammation, characterized by an increase in oxidative stress markers (e.g., 8-Hydroxyguanosine (8-OHdG), malondialdehyd (MDA)), acute phase proteins (e.g., c-reactive protein (CRP)), prostaglandins (PG) (e.g., PGE2), or cytokines, such as interleukins (IL) (e.g., IL-1β, IL-1α, IL-6, IL-8), and tumor necrosis factors (TNF) (e.g., TNF-α) [4]. Additionally, systemic inflammation stimulates neutrophils, immune cells that are part of the innate immune defense and serve to identify and destroy microorganisms, to release their enzymes, such as matrixmetalloproteinases (MMPs) [4,5], myeloper-oxidase (MPO), and neutrophil elastase (NE) [6]. Thus, microorganisms and their components found in the oral cavity as well as in inflammatory mediators may disseminate in the body, invade the fetal–placental unit, consequently inducing an inflammatory response, or circulate to other organs, increasing systemic inflammation through acute phase protein responses, which may later enter the fetal–placental unit [7,8]. As a result, this may lead to adverse pregnancy outcomes such as preterm birth, low birthweight (LBW), preeclampsia (PE), pregnancy-induced hypertension (PIH) or early pregnancy loss (EPL) [9]. In this regard, research has shown that periodontitis, a polymicrobial biofilm-induced inflammatory disease, has been associated with adverse pregnancy outcomes in the past [10,11]. Thus, understanding the microbiology and immunology of oral conditions in pregnancy is essential.

Inflammatory processes during pregnancy are also influenced by dietary choices, making nutrition during pregnancy an important contributor to general and oral health in the mother and child [12]. Multiple factors, such as nutritional deficiencies or snacking habits, lead to tooth decay. Cravings for sweets and fast foods that increase acidity in the saliva and affect the microbial composition in the oral cavity, possibly favoring a pathogenic flora, have been described by research in recent years and warrant a focus on nutrition when talking about the improvement in oral health during pregnancy [5,13,14,15].

A recent systematic review by Jang and colleagues [5] reported on the presence and abundance of microorganisms in the oral cavity during pregnancy as well as the distinct patterns between pregnancy stages, which adds to the knowledge that oral bacteria and their metabolites may be linked to or serve as biomarkers for certain systemic diseases [16]. However, while DNA sequencing for oral bacterial identification has previously been studied, the response and changes of inflammatory biomarkers in the oral biofilm and their association with pregnancy outcomes are yet to be understood.

**Figure 1:**
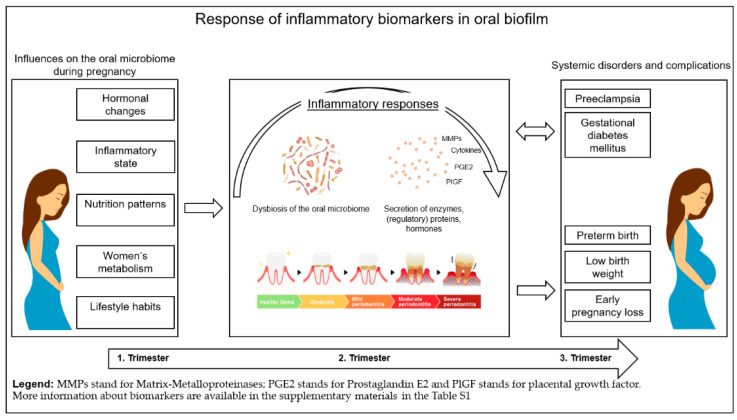
Response of inflammatory biomarkers in oral biofilm.

Highlighting the importance of oral health during pregnancy, we aimed to understand inflammatory responses in the oral biofilm during pregnancy, as well as its association with maternal health, by identifying biomarker patterns in saliva or gingival crevicular fluid (GCF) that may serve as early markers of pregnancy-related complications. We conducted a systematic review of the available literature to assess: (1) inflammatory responses in the oral biofilm during pregnancy, (2) the association between inflammatory responses in the oral biofilm during pregnancy and maternal, oral or systemic conditions, (3) changes in the response of inflammatory biomarkers found in the oral biofilm during different pregnancy stages, and (4) the value of other risk factors such as nutrition patterns, lifestyle habits, hormonal changes, or weight gain. Figure 1 gives an overview of the inflammatory response in oral biofilm during pregnancy. In the beginning of pregnancy, multiple factors can influence the composition of the oral microbiome, which in turn affects oral health. Oral dysbiosis and poor dental health trigger the production of inflammatory mediators in the oral cavity, indirectly affecting the fetal–placental unit and leading to adverse pregnancy outcomes.

## 2. Materials and Methods

This review followed the Preferred Reporting Items for Systematic Reviews and Meta-Analyses (PRISMA) guidelines [17]. It was prospectively registered on the National Institute for Health Research International Prospective register of Systematic Reviews (PROSPERO) (ID: CRD42022308361), available from https://www.crd.york.ac.uk/prospero/display_record.php?ID=CRD42022308361 (accessed on 9 April 2022).

### 2.1. Literature Search Strategy

Database searches were conducted in December 2021 and updated in April 2022 to identify the inflammatory response in oral biofilm during pregnancy. Searches were conducted within the following databases: PubMed, Web of Science (Core Collection) and Cochrane Library. The search strategy was based on a clear and careful selection of key words and terms. After repeated attempts and adjustment, the final search strategy was built (Table S1). The search filter was set on human beings and language was restricted to English and German. Only articles published from date of inception until 30 April 2022 were included. In addition, supplementary searches were performed by hand of reference lists of relevant views or included studies (backward search). References from reviews and systematic reviews were also checked manually for further screening.

### 2.2. Inclusion and Exclusion Criteria

The following eligibility criteria were considered according to the PICOS framework:

Types of participants: all pregnant women of reproductive age (healthy or non-healthy), independent of gestational age (GA).

Type of intervention/phenomena of interest: pregnancy.

Types of comparisons: changes in inflammatory biomarkers found in the oral biofilm during pregnancy; changes in periodontal status during pregnancy; differences in inflammatory responses in oral biofilm between pregnant vs. non-pregnant women and healthy pregnant vs. diseased pregnant women or pregnant women with complications.

Outcomes: inflammatory response in the oral biofilm; association between the inflammatory responses in oral biofilm, the maternal, oral or systemic conditions and the periodontal status. The latter was included as an important inclusion criterion to assess the subject’s oral health status, seeing as it helps the authors to assess whether the classification of subjects into periodontitis cases and controls was performed correctly, and to understand how the inflammatory response works in periodontitis patients.

Types of studies: case–control studies, cross-sectional studies, retrospective and prospective cohort studies, randomized and non-randomized controlled trials.

Excluded: studies with only DNA sequencing for bacterial identification, studies that did not perform a periodontal examination among their study participants or collected biofilm samples other than in the saliva or GCF, as well as studies without record of relevant inflammatory biomarkers. Furthermore, we excluded in vitro studies, animal studies, literature reviews, letters to the editor, editorials, conference abstracts, patient handouts, case reports or case series, patents, papers with an abstract only, book chapters, or trial registrations.

### 2.3. Data Extraction and Screening

Search results of all databases were merged, and duplicate records were identified and eliminated using the reference management tool EndNote 20.2.1. The titles and abstracts of all studies were scanned by three reviewers, and studies against the inclusion criteria or within the exclusion criteria were removed. Questions and disagreements were presented at regular meetings and resolved through discussion. Further screening of studies was conducted by reading the full text of the studies according to the criteria. If a study was not included, the reasons for exclusion were reported. Disagreements about exclusion criteria or the eligibility of an article were resolved according to the majority principle or until a consensus was reached.

A predetermined grid was adopted to perform data extraction, including the following information: author, publication year, country, study design, study population, measurement interval, periodontal examination, sample collection method, sample analysis, evaluated biomarker, and main study findings. This step was completed by two reviewers through full-text reading. Uncertainties were discussed and all contents were approved by three reviewers before inclusion in the final table.

To define the subject’s periodontal health, examination included the assessment of some or all of the following parameters: pocket probing-depth (PPD), bleeding on probing (BOP), calculation of the clinical attachment loss (CAL), and reporting of the plaque index (PI). Other clinical indices reported to assess oral health, more specifically plaque accumulation or gingival bleeding tendency, included the approximal plaque index (API), gingival bleeding index (GBI), sulcus bleeding index (SBI) as well as the DMFT-index, which stands for the number of decayed, missing, and filled teeth. Higher parameters and indices indicate periodontal disease progression.

For saliva samples, with a participant’s preparation prior to sample collection, the sample collection method as well as the processing and storage of the sample were reported. Similarly, we reported the collection method of GCF samples, including instrument, dental sites and collection time, as well as the storage and elution process of the sample.

The screening of full texts revealed substantial heterogeneity in study population and evaluated biomarkers, which precluded conducting a meta-analysis. The differences in the same parameters of interest measured were too broad for a meaningful meta-analysis. Thus, a narrative synthesis was conducted instead.

### 2.4. Quality Assessment

The quality of the selected articles was assessed depending on the type of study. For intervention studies, the Effective Public Health Practice Project Quality Assessment Tool for Quantitative Studies (EPHPP) [18] was used. Each study was evaluated according to six components: selection bias, study design, confounders, blinding, data collection methods, withdrawals and drop-outs. Each component was given a rating of ‘strong’, ‘moderate’ or ‘weak’, in addition to an overall rating of study quality (global rating) based on the component ratings. A ‘strong’ global rating was assigned when no component was rated as weak, a ‘moderate’ global rating indicated that one component was rated as ‘weak’, and a ‘weak’ global rating was assigned when more than one component was rated as ‘weak’. For cohort and cross-sectional studies, the quality assessment tool for observational cohort and cross-sectional studies of the National Heart, Lung, and Blood Institute was used [19]. The studies were assessed according to 14 criteria. Each criterion was evaluated and given a response of ‘yes’ (the criteria was met) or ‘no’ (the criteria was not met). The overall rating of study quality (good, fair, or poor) was based on the meeting of criteria and overall study quality, as assessed by the reviewer. The risk of bias was assessed by at least two reviewers independently. If agreement was not achieved, a third reviewer would contribute to the assessment and differences were resolved through discussion. All studies were included in the narrative synthesis.

## 3. Results

**Figure 2:**
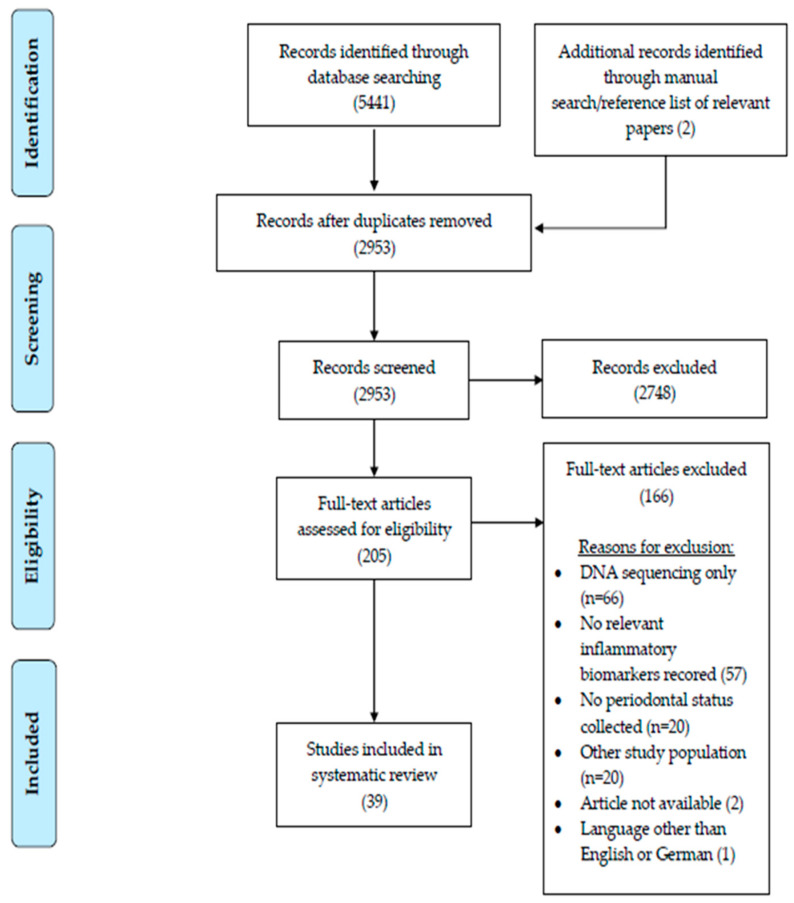
Flow diagram of study identification.

A total of 2953 articles were assessed after duplicates were removed. The PRISMA flow diagram was used to report the study selection process (Figure 2). From the remaining 2953 records, 2748 were excluded after title and abstract screening. The remaining 205 studies proceeded to a full text review; 166 studies were eliminated based on the exclusion criteria and a final 39 articles were chosen for qualitative assessment. 

### 3.1. Study Characteristics

A total of 39 studies are categorized into the following subgroups: 22 studies on the association between inflammatory responses in oral biofilm and multi-systemic disorders or health conditions during pregnancy (Table 1); nine studies on inflammatory responses in oral biofilm between healthy pregnant and non-pregnant women (Table 2); six studies on changes in the response of inflammatory biomarkers in oral biofilm between pregnancy stages (Table 3); two studies on inflammatory responses in oral biofilm between pregnant and non-pregnant women with periodontitis (Table 4). Five intervention studies and 34 observational studies were found. 23 studies were cross-sectional and all of them included at least one comparison group. Among the longitudinal studies, eight compared more than one cohort over time. In all 39 studies, the periodontal examination was performed separately from the sample collection of saliva or GCF. Of the 26 studies that collected saliva samples from among its study participants for analysis, of which five studies collected both saliva as well as GCF samples, the most commonly reported collection method was the passive drooling technique or spitting or expectorating into a tube. In most cases, participants were asked to fast overnight or to refrain from food or any beverages for at least one hour before sample collection. In the 18 studies that collected GCF samples for analysis of inflammatory responses, paper strips were used to collect samples in the sulcus. The majority of samples were eluted in buffered saline, mostly phosphate, and then immediately frozen. Three quarters of the studies used an enzyme-linked immunosorbent assay (ELISA) to analyze the saliva or GCF samples. Sample sizes of the studies ranged from 19 to 226 participants. All characteristics of the included studies are summarized in Table 1, Table 2, Table 3 and Table 4. Quality assessment for interventional as well as cohort and cross-sectional studies are included in the last column of all tables.

### 3.2. Association between Changes in Inflammatory Responses in the Oral Biofilm and Multisystemic Disorders or Health Conditions during Pregnancy (Table 1)

In total, 22 studies examined changes in inflammatory responses in oral biofilm and multi-systemic disorders or health conditions during pregnancy.

#### 3.2.1. Gestational Diabetes Mellitus

Six cross-sectional studies compared differences in inflammatory response in oral biofilm between pregnant women with gestational diabetes mellitus (GDM) and pregnant women without GDM [20,21,22,23,24,25]. The detected cytokine levels (IL-10, IL-8, IL-6, TNF-R1/2, monocyte chemoattractant protein-1 (MCP-1)) [20,21,23] were significantly higher in the GDM compared to the non-GDM group. Furthermore, greater protein concentrations of vascular endothelial growth factor (VEGF), soluble intercellular adhesion molecule-1 (sICAM-1), and placental growth factor (PIGF) were found in the GDM group [22,23]. Zygula and colleagues [25] specifically compared oxidative stress markers between diet-controlled and insulin-controlled women with GDM. Salivary MDA levels among women with GDM that were diet-treated were found to be lower compared to those of the insulin-treated subjects.

#### 3.2.2. Periodontitis

A total of five studies collected saliva samples to compare the inflammatory responses in oral biofilm between pregnant women with gingivitis or periodontitis, and healthy pregnant or non-pregnant women [26,27,28,29,30]. In comparison to healthy pregnant women, women with periodontitis demonstrated significantly higher IL-17 levels [29]. Similarly, significantly higher IL-1β and MMP-8 levels were also found in women with gingivitis compared to healthy pregnant women [30]. Moreover, Machado and colleagues [28] showed significantly higher TNF-α and IL-6 levels among pregnant women with periodontitis compared to healthy non-pregnant controls. Differences in inflammatory biomarker levels between stages of periodontitis (moderate vs. severe) were, however, not significant. Studying the salivary proteome, Balan and colleagues [26] found significantly lower levels of cystatins SA, SN, and C in pregnant women with gingivitis compared to healthy non-pregnant women, indicating a lack of protection of oral tissues from proteolytic enzymes. Differences between the gingivitis groups and healthy pregnant women were not significant. Only one study population was differentiated more closely according to BMI and socioeconomic status [29].

#### 3.2.3. Preeclampsia

Three cross-sectional studies compared normotensive women and women with PE [31,32,33]. The authors found significantly higher PPD and CAL scores, a significantly higher concentration of PGE2, TNF-α, and IL-1β [31] as well as higher levels of placental alkaline phosphatase (PLAP) and soluble fms-like tyrosine kinase-1 (SFlt-1), which bind the angiogenic factors VEGF and PlGF, in the PE group compared to normotensive women [32]. Canakci and colleagues [33] compared normotensive women with and without periodontitis with preeclamptic women with and without periodontitis. Superoxide dismutase and glutathione peroxidase (GPx) were lowest in preeclamptic women with periodontal disease, and MDA levels were highest in this group. All three studies provide information on the weight of the subjects, but only one study relativizes this to height (BMI) and mentions further metabolic diseases as an exclusion criterion [32].

#### 3.2.4. Obesity

A prospective cohort study from Brazil [34] and a cross-sectional study from Italy [35] looked at oral biofilm activities in obese women compared to pregnant women with normal weight. Obesity was defined as having a body mass index (BMI) ≥ 30.00 kg/m^2^. While Foratori and colleagues [34] defined GDM as an exclusion criterion, half of the obese pregnant group among study participants described by Zambon and colleagues had GDM [35]. In both studies, saliva samples were collected during the 3rd trimester and authors found a higher prevalence of periodontitis as well as significantly higher levels of TNF-α, IL-1β, total antioxidant capacity (s-TAC) and CRP, particularly when GDM was diagnosed. Moreover, a significant interaction effect between maternal BMI and periodontitis was found for s-TAC levels.

#### 3.2.5. Other Pregnancy Complications

Differences in inflammatory response in oral biofilm among pregnant women with other pregnancy complications, such as EPL or premature birth, were discussed in six studies [36,37,38,39,40,41]. A cross-sectional study from Italy reported worse periodontal status among preterm LBW mothers compared to healthy controls [36]. Moreover, GCF, PGE2 and IL-1β levels were significantly higher in the preterm LBW mothers. In comparison, the cross-sectional case–control study from Nikolic and colleagues [37] found no significant differences in salivary PGE2 and IL-1β between preterm delivery women and women that delivered at term. Furthermore, no significant differences in periodontal parameters were found among German women with high risk for a preterm LBW infant [38]. Although the case group reported higher IL-1β levels compared to the controls, the results were not significant. In a prospective case–control study analyzing women with preterm premature rupture of membranes (PPROM), a risk factor for preterm birth, significantly higher periodontal inflammation was reported among the PPROM group [39]. During the second and third trimesters, the PPROM group showed significantly lower IL-8 and CRP levels as well as higher IL-10 levels compared to the test group. Furthermore, IL-1β levels significantly increased between the third trimester and after delivery among the test group. A cross-sectional study from Poland examined differences in oxidative stress levels for women with PIH and women with intrauterine growth restrictions (IUGR) as well as uncomplicated pregnancies [40]. While no differences were found between the PIH and control group with regard to inflammatory biomarkers found in saliva, salivary aldehyde dehydrogenase (ALDH) activity and oxygen radical absorbance capacity (ORAC) concentration were higher in the IUGR group compared to the others. Furthermore, the authors also collected dietary data from their study participants using a standardized food frequency questionnaire (FFQ). No dietary differences were found between the group concerning energy, fiber, flavonoids, vitamin C or iron.

**Table 1:** Study groups with healthy pregnant and diseased pregnant women.

Author (year)	Country, Study Design	Investigated Groups *(n of Subjects)*	Measurement Interval	Periodontal Examination	Sample Collection	Sample Analysis	Evaluated Biomarkers	Main study Findings and Key Messages	Quality Assessment
Balan et al. (2021a) [26]	Singapore, cross-sectional study	**Discovery dataset:** Pregnant with gingivitis (10) Pregnant without gingivitis (10) Non-pregnant healthy controls (10) **Validation dataset:** Pregnant with gingivitis (16) Pregnant without gingivitis (16) Non-pregnant healthy controls (16)	One time-point: Week 21–28 GA; day 12–14 of menstrual cycle for non-pregnant women	PPD, BOP, PI, GBI	*Saliva:* **Preparation:** Subjects provided samples after thorough rinsing of the mouth and had refrained from food or drink for at least 1 h before collection **Method:** Passive drooling technique **Processing:** Protein precipitation using TCA at 4 °C **Storage:** Collected supernatants stored at −80 °C	iTRAQ, ELISA	Salivary cystatins, Protein profiles	- Pathways associated with neutrophil-mediated immune response and antioxidant defense mechanism are significantly higher in pregnant women with gingivitis than non-pregnant healthy controls	*Fair*
Balan et al. (2021b) [41]	Singapore, cross-sectional case–control	**Pregnant (20)** Early pregnancy loss (10) Healthy pregnant (10)	One time-point	BOP, PI, DMFT	*Saliva:* **Preparation:** Subjects refrained from food or drink for at least one hour before sample collection **Method:** not specified **Processing:** Centrifuged at 2000 g for 15 min at 4 °C **Storage:** Supernatant was treated with TCA and stored at 4 °C	iTRAQ, LC-MS, ELISA	Protein profiles	- The cellular process is significantly affected in subjects with early pregnancy loss	*Fair*
Balan et al. (2022) [27]	Singapore, cross-sectional study	**First sample set:** Pregnant (24) **Second sample set:** Pregnant with gingivitis (10) Pregnant without gingivitis (10) Non-pregnant (10)	One time-point: Week 20–35 GA	BOP, PI, GBI	*Saliva:* **Preparation:** Subjects provided samples after thorough rinsing of the mouth and had refrained from food or drink for at least 1 h before collection **Method:** Spitting method **Processing:** Centrifuged at 2000 rpm for 15 min at 4 °C **Storage:** not specified	iTRAQ, ELISA, SEM	Protein profiles, NEs, MPOs, Nucleosomes	**Salivary proteome shifts during pregnancy:** - Upregulated defense and humoral responses during the first trimester. - Decrease in immune response during the second and third trimester - - NETs formation is increased significantly in the third trimester.	*Good*
Canakci et al. (2007) [33]	Turkey, cross-sectional study	**Pregnant women (40)** Preeclampsia and periodontitis (10) Preeclampsia without periodontitis (10) Normotensive and periodontitis (10) Normotensive without periodontitis (10)	One time-point	PPD, CAL, BOP	*GCF: Preparation?* **Method:** Filter paper strips **Site:** Mesio-buccal and disto-palatal site on each tooth **Collection time:** 30 s. **Processing:** Eluted in phosphate-buffered saline **Storage:** Stored at −80 °C *Saliva:* **Preparation:** Subjects provided samples after an overnight fast **Method:** Expectorating into disposable tubes **Processing:** Centrifuged at 1000 rpm for for 10 min at 4 °C **Storage:** Supernatants stored at −80 °C	Assay for superoxide dismutase	GPx and superoxide dismutase, MDA, TAC	**Preeclamptic women:** - Total antioxidant capacity in saliva and GCF are lowest in preeclamptic women with periodontal disease	*Fair*
Carta et al. (2004) [36]	Italy, cross-sectional study	**Pregnant (92)** Premature LBW (46) Uncomplicated pregnancy (46)	One time-point	PPD, BOP, PI, DMFT	*GCF:* **Site:** not specified **Method:** Paper strips Collection time: not specified **Processing:** not specified **Storage:** Stored in liquid nitrogen	ELISA	IL-1β, PGE2	- Mean GCF-IL-1β and PGE2 levels were significantly higher in premature LBW - Mothers with higher PGE2 levels had smaller and more premature babies.	*Poor*
Chaparro et al. (2016) [32]	Chile, cross-sectional study	**Pregnant (30)** Preeclampsia (10) Normotensive (20)	One time-point	PPD, CAL, BOP	*Saliva:* **Preparation:** not specified **Method:** not specified **Processing:** Centrifuged at 1500× *g* for 10 min at 4 °C **Storage:** Collected supernatants stored in liquid nitrogen *GCF:* **Method:** Filter paper strips **Site:** not specified Collection time: 30 s. **Processing:** Eluted in 100 µL phosphate-buffered saline **Storage:** Stored in liquid nitrogen	ELISA, quantitative real-time poly-merase chain reaction	PLAP, PIGF, sflt1, EV-Cd63+	**Preeclamptic women:** - Significantly higher PLAP concentration & PLAP/CD63+ ratio in GCF - - higher sFlt-1 concentrations in saliva and GCF	*Fair*
Chaparro et al. (2018) [22]	Chile, nested case–control with a prospective cohort	**Pregnant (226)** With GDM (14) Without GDM (212)	One time-point: Week 11–14 GA	PPD, CAL, BOP	*Saliva:* **Preparation:** not specified **Method:** not specified **Processing:** not specified **Storage:** Collected supernatants stored in liquid nitrogen *GCF:* **Method:** Filter paper strips **Site:** Four periodontal pockets (1 × quadrant) at the most affected periodontal site Collection time: 30 s. **Processing:** Eluted in 100 µL phosphate-buffered saline **Storage:** Stored in liquid nitrogen	ELISA	PlGF and sFlt-1	**Women with GDM:** - Significantly greater BOP, PD, CAL & periodontal inflamed surface area - Significantly greater initial glycemia and GCF-PlGF concentrations - - Associations between glycemia and GCF-PlGF	*Good*
Foratori et al. (2021) [34]	Brazil, prospective cohort study	**Pregnant (50)** Obese (25) Normal weight (25)	**T1:** Week 27–36 GA **T2:** 3 months postpartum	PPD, CAL BOP	*Saliva:* **Preparation:** Subjects were instructed to not consume any food or drink and to brush their teeth before the appointment **Method:** Subjects rinsed their mouths with 5 mL of deionized water. Afterwards, they were instructed to chew a piece of sterile rubber (0.5–1.0 cm of latex tube) attached to a 30 cm piece of dental floss. Stimulated saliva was collected in 5 min **Processing:** Saliva samples were lyophilized **Storage:** Collected supernatants stored at −80 °C	Luminex xMAP	IL-1β, TNF-α, leptin	**Pregnant obese women:** - Higher prevalence of periodontitis in T1 and T2 - Higher salivary levels of TNF-α and IL-1β in T1. - Significantly decreased IL1ß after delivery - Children with lower birth weight - - No differences for leptin levels measurable between both groups but significant reduction of leptin levels between pregnancy periods	*Fair*
Gümüs et al. 2014 [21]	Turkey, cross-sectional study	**Pregnant (167)** GDM with gingivitis (71) GDM without gingivitis (390) Non-GDM with gingivitis (38) Non-GDM without gingivitis (28)	One time-point: Week 24–28 GA	PPD, CAL, BOP	*Saliva:* **Preparation:** Overnight fast during which subjects were requested not to drink (except water) or chew gum **Method:** Expectorating into polypropylene tubes **Processing:** Centrifuged at 13,000× *g* for 5 min at 4 °C **Storage:** Collected supernatants stored at −40 °C *GCF:* **Method:** Filter paper strips **Site:** Buccal aspects of two interproximal sites in single-rooted teeth **Collection time:** 30 s. **Processing:** Eluted in 1 mL phosphate-buffered saline **Storage:** Stored at −40 °C	ELISA	IL-6, IL-8, RANKL, OPG, APRIL, BAFF	**Women with GDM:** - higher IL-8, saliva sRANKL levels **Women with GDM + gingivitis:** - significantly higher biomarker level than orally healthy women with GDM saliva	*Fair*
Machado et al. (2018) [28]	Portugal, cross-sectional study	**Pregnant (44)** Without periodontitis (15) Mild/moderate periodontitis (16) Severe periodontitis (13)	One-time-point	PPD, CAL	*Saliva:* **Preparation:** not specified **Method:** Passive drooling technique **Processing:** not specified **Storage:** Collected supernatants stored at −80 °C	ELISA	TNF-α, IL-6	- Women with periodontitis exhibit significantly higher levels of salivary IL-6 and TNF-α	*Fair*
Mahilkar et al. (2021) [29]	India, case–control study	**Pregnant (40)** With periodontitis (20) Without periodontitis (20)	**T1:** 2nd trimester **T2:** postpartum	PPD, CAL, BOP, PI	*Saliva:* **Preparation:** Subjects were asked to refrain from eating or drinking for 2 h prior to saliva collection **Method:** Passive drooling technique **Processing:** Centrifuged at 2500 rpm for 10 min at 4 °C **Storage:** Collected supernatants stored at −80 °C	ELISA	IL-17	- IL-17 levels in saliva were significantly higher in pregnant women with periodontitis - No association between Il-17, periodontitis, cases of preterm deliveries and low birth weight	*Fair*
Nikolic et al. (2020) [37]	Serbia, cross-sectional	**Pregnant (112)** Preterm birth (56) Term delivery (56)	One time-point:Within 48 h following delivery	PD, CAL, BOP, PI	*Saliva:* **Preparation:** No antiseptic mouth rinse was used prior to collection **Method:** not specified **Processing:** Centrifuged at 3500 rpm for 20 min at 4°C **Storage:** Two thirds of supernatant were stored at −70 °C, one third of supernatant was stored at −20 °C	ELISA	IL-1β PGE2	- Significant association between PGE2 and IL-1ß in preterm birth group	*Fair*
Noack et al. (2005) [38]	Germany, cross-sectional study	**Pregnant (101)** Preterm LBW (59) Term delivery (42)	One time-point	PPD, CAL, BOP, PI	*GCF:* **Method:** Filter paper strips **Site:** Mesio-vesibular of each first or second molar **Collection time:** not specified **Storage:** Stored at −20 °C Elution/Processing: not specified	ELISA	IL-1β	- No significant differences in any aspects of the studied periodontitis parameters	*Fair*
Oettinger-Barak et al. (2005) [31]	Israel, cross-sectional study	**Pregnant (30)** Preeclampsia (15) Normotensive (15)	One time-point: 48 h before delivery	PPD, CAL, PI, GBI	*GCF:* **Method:** Periodontal paper strips **Site:** Periodontal sulci and pockets of the Ramfjord index teeth **Collection time:** 30 s. **Processing:** Insertion into an individual sterile tube containing 1.0 cc distilled water; Stand at room temperature for 30 min and shaken every 5 min to facilitate extraction of the sample **Storage:** Stored at −70 °C	ELISA	IL-1β, IL-6, TNF-α, PGE2 +	**Preeclamptic women:** - Significantly higher PPD and CAL scores - Significantly higher PGE2, TNF-α, IL-1β levels - - No differences in PI, GI, and mean gingival overgrowth scores	*Fair*
Ozcaka et al. (2016) [20]	Turkey, cross-sectional study	**Pregnant (161)** With GDM (96) Without GDM (65)	One time-point: 24–28 weeks GA	PPD, BOP, PI	*GCF:* **Method:** Paper strips Site: not specified **Collection time:** not specified **Processing:** Eluted in 0.5 mL phosphate-buffered saline **Storage:** Stored at −40 °C	ELISA	IL10, TNF-α, IL-33	**Women with GDM:** - significantly higher PI, BOP values - - significantly higher IL-10 concentrations and total amounts	*Fair*
Stadelmann et al. (2015) [39]	Switzerland, prospective case–control study	**Pregnant (56)** Preterm premature rupture of membranes (PPROM) (32) Uncomplicated pregnancy (24)	**T1:** Week 20–35 GA **T2:** within 48 h after parturition **T3:** 4–6 weeks after parturition	PPD, CAL, BOP, PI	*GCF:* **Method:** Paper strips **Site:** Mesiobuccal site of each first molar in all quadrants **Collection time:** 15 s. **Processing:** Eluted in 750 μL phosphate-buffered saline containing proteinase inhibitor **Storage:** Stored at−80 °C	ELISA	IL-1β, CRP, IL-10, CRP	**Women with PPROM:** - Higher periodontal inflammation, decreasing over time in both groups - lower GCF levels of IL-8 and CRP - higher IL-10 levels - - at T2 no different IL-1β levels between groups	*Fair*
Surdacka et al. (2011) [23]	Poland, cross-sectional	**Pregnant (63)** Diabetes (30) Healthy (33)	One time-point: 1st trimester	PPD, CAL, API, GBI, SBI	*Saliva:***Preparation:** Subjects were asked to rinse their mouths thoroughly with water. Saliva was collected at least 2 h after meal. **Method:** Passive drooling technique **Processing:** Centrifuged at 10,000× *g* for 5 min at 4 °C **Storage:** Supernatant stored at −30 °C	Elmman Test, Lowry method, TAS kit, Bioxytech assays, DuoSet Immunoassays	TP, SOD activity, Catalase activity, UA, Free -SH groups, TAC, MCP-1, GRO-α, IL-8, CSF, IL-6, IL-6sR, TNFα, TNF-R1, TNF-R2, IL-17, HGF, SDF-1, VEGF, sICAM-1	**Women with GDM:** - Significantly higher protein concentrations and increased antioxidant capacity - - Considerably higher concentrations of MCP-1, IL-8, IL-6sR, TNF-R1/2, VEGF and sICAM-1	*Poor*
Yang et al. (2019) [30]	USA, cross-sectional pilot study	**Pregnant (34)** With Gingivitis (12) Without gingivitis (22)	One time-point: 3rd trimester	GBI	*Saliva:* **Preparation:** not specified **Method:** not specified **Processing:** not specified **Storage:** Collected supernatants stored at −80 °C	ELISA	IL-1β, MMP-8, CRP	- Women in the gingivitis group have significantly higher levels of IL-1β and MMP-8 - CRP levels did not significantly differ. - Moderate to strong positive correlations between all three inflammatory markers (IL-1β, MMP-8, CRP). - - No relationship between gestational age at birth and any of the inflammatory markers or α -diversity.	*Fair*
Zalewska et al. (2013) [24]	Poland, cross-sectional study	**Pregnant (50)** With GDM (25) Without GDM (25) **Non-pregnant (25)**	One time-point	GBI, DMFT	*Saliva:* **Preparation:** Subjects were asked to refrain from food and beverages, except water, for one hour before saliva collection **Method:** Spitting method **Processing:** Centrifuged at 3000× *g* for 20 min at 4 °C **Storage:** Supernatant stored at −80 °C	Marciniak method, Zwierz method, Lowrys’s method, Spectrophotometry	HEX, GAL, MAN, FUC, GLU	**Women with GDM:** - Specific activities of exoglycosidases	*Fair*
Zambon et al. (2018) [35]	Italy, cross-sectional study	**Pregnant (62)** Normal weight (27) Obese (35)	One time-point: 3rd trimester	PPD, CAL, BOP, PI	*Saliva:* **Preparation:** not specified **Method:** Passive drooling technique **Processing:** Centrifuged at 4000× *g* for 10 min at 4 °C Storage: not specified	Antioxidant Assay kit, ELISA	s-TAC, s-CRP	**Pregnant obese women:** - Significantly higher levels of s-TAC, s-CRP, and p-CRP particularly in the presence of GDM - - With periodontitis significant increase in local and systemic parameters	*Fair*
Zygula et al. (2019) [25]	Poland, cross-sectional study	**Pregnant (89)** With GDM (59) Without GDM (30)	One time-point	PPD, CAL, PI, GBI	*Saliva:* **Preparation:** Subjects fasted overnight (at least 6 h) **Method:** Spitting method **Processing:** Centrifuged at 10,000× *g* for 10 min **Storage:** Supernatant stored at −80 °C	Fluorometric method for ALDH, ORAC-fluorescein fluorometric assay, MDA Assay	ALDH, ORAC, MDA	Women with GDM: - Elevated concentration of salivary MDA - inactivation of ALDH - -Tendency for lower ORAC	*Fair*
Zygula et al. (2020) [40]	Poland, cross-sectional study	**Pregnant (104)** Pregnancy-induced hypertension (PIH) (27) Intrauterine growth restriction (IUGR) (30) Uncomplicated pregnancy (47)	One time-point	PPD, CAL, PI, GBI	*Saliva:* **Preparation:** Subjects fasted overnight (at least 6 h) **Method:** Spitting method **Processing:** centrifuged at 10,000× *g* for 10 min **Storage:** Supernatant stored at −80 °C	Fluorometric method for ALDH, ORAC-fluorescein fluorometric assay, MDA Assay	ALDH, ORAC, MDA	**IUGR group:** - Increased concentration of ORAC and higher activity of salivary ALDH - ORAC in saliva and plasma correlate **Diet:** - Surveyed with FFQ, no dietary differences between the group concerning energy, fibre, flavonoids, vitamin C or iron.	*Fair*

### 3.3. Differences in Inflammatory Responses in Oral Biofilm between Healthy Pregnant and Non-Pregnant Women

Considerable differences in inflammatory biomarkers found in the oral biofilm were seen in pregnant women in comparison to non-pregnant women. We identified three studies that examined cytokine levels in GCF. A prospective case–control study from China [42] found significantly higher IL-1β levels among pregnant compared to non-pregnant women. The other two studies found no significant difference in Il-10, IL-1β or IL-6 levels when comparing the two groups [43,44]. After implementation of a non-surgical periodontal therapy intervention, which includes removal of subgingival dental plaque and planning of the exposed surfaces of the root, IL-1β significantly decreased in the non-pregnancy group, but no statistical difference was determined for GCF IL-1β in the pregnant group [43]. Interestingly, at the end of periodontal therapy, Yarkac and colleagues [44] reported increasing IL-6 levels among the pregnant group, while the non-pregnant group demonstrated decreasing IL-6 levels, which they attribute to alterations in the immune system and hormonal state during pregnancy.

Enzyme activity of MMP-8 was examined by three studies. A cross-sectional study from Germany [45] reported higher MMP-8 concentration in the pregnant vs. the non-pregnant group. Although Gürsoy and colleagues [46,47] found no differences in MMP-8 levels, they reported significantly higher MMP-8 concentrations right after delivery. Compared to non-pregnant women, salivary lipid peroxidation (LPO) and total siliac acid (TSA) levels increased during the second trimester of pregnancy and were significantly higher [48]. In a case–control study of Turkish pregnant women in the second or third trimester and healthy non-pregnant controls, no significant differences in salivary Thiobarbituric acid reactive substances (TBARS), a byproduct of lipid peroxidation, were detected [49]. However, significantly lower 8-OHdG and higher GPx levels were detected in non-pregnant women compared to pregnant women. With regard to defensins, Gürsoy et al. [50] reported significantly lower hBD-1 and HNP-1 concentrations during the third trimester compared to the non-pregnant groups.

When comparing periodontal parameters between pregnant and non-pregnant women, the majority of studies did not find significant differences between the groups for most of the examined parameters [42,43,44,48]. Because periodontal parameters were collected at different stages during pregnancy, it is difficult to compare the studies or draw consistent conclusions. However, in terms of changes during pregnancy, three studies reported deeper pockets depths and increased gingival inflammation as the pregnancy progressed, specifically comparing the first and second trimester [42,46,47]. Increased periodontal parameters reflect periodontal disease progression. After application of a non-surgical periodontal therapy intervention, periodontal parameters significantly decreased among pregnant women as well as non-pregnant women [43,44]. However, while the two groups did not differ significantly in their periodontal parameters prior to the therapy, the pregnant women exhibited significantly greater PPD and greater gingival inflammation than the non-pregnant women after periodontal therapy. The authors assume that this may be due to the women’s response to increasing stress levels during pregnancy.

**Table 2:** Differences in inflammatory responses in oral biofilm between healthy pregnant and non-pregnant women.

Author (year)	Country, Study Design	Investigated Groups (n of Subjects)	Measurement Interval	Periodontal Examination	Sample Collection	Sample Analysis	Evaluated Biomarker	Main study Findings and Key Messages	Quality Assessment
Ehlers et al. (2013) [45]	Germany, cross-sectional study	Pregnant (20) Non-pregnant (20)	One time-point	PPD, CAL, SBI, PI	*GCF:* **Method:** Filter paper strips Site: Gingival sulcus of all four first molars **Collection time:** 30 s. **Processing:** Eluted in 800 μL of HEPES buffer **Storage:** not specified	DentoAnalyzer	aMMP-8	- Pregnant women showed significantly increased probing pocket depths - Gingival inflammation was present in 80% of the pregnant women, but only in 40% of the control subjects. - Higher aMMP-8 values in pregnant women	*Fair*
Gümüs et al. (2015) [49]	Turkey, case–control study	Pregnant (115) Non-pregnant (72)	**T1:** 1st or 2nd trimester **T2:** 6 months postpartum	PPD, CAL, BOP, PI	*Saliva:* **Preparation:** Subjects were asked to fast overnight and to rinse their mouth with tap water prior to sample collection **Method:** Expectoration into polypropylene tubes for 5 min **Processing:** Centrifuged at 10,000× *g* for 15 min at −4 °C **Storage:** Collected supernatants stored at −80 °C	ELISA, GPx assay kit, TBARS Parameter Assay Kit	GPx activity, 8-OHdG, TBARS	**Oxidative stress markers:** - Clinical measurement of periodontal disease severity correlate only in the postpartum and the non-pregnant group - No correlations with TBARS in the pregnant and postpartum group, but significant reduction of TBARS after birth **Pregnant women:** - Levels of 8-OHdG significantly elevated - Significantly lower activity of antioxidant enzyme GPx	*Good*
Gürsoy et al. (2010a) [46]	Finland, prospective cohort study	Pregnant (30) Non-pregnant (24)	**T1:** Week 12 GA **T2:** Week 14 GA **T3:** Week 25–27 or Week 34–38 GA **T4:** 4–6 Weeks postpartum **T5:** after breastfeeding	PPD, CAL, BOP, PI	*GCF:* **Method:** Filter paper strips **Site:** Mesiobuccal sites of all first molars or, if missing, second molars **Collection time:** 30 s. **Processing:** Eluted in 75 mL of 50 mM Tris-HCl (pH 7.8), including 0.2 M NaCl and 1 mM CaCl2 **Storage:** Stored at −20 °C	ELISA	MMP-8, elastase, MPO, TIMP-1	**Pregnant women:** - Significantly increased BOP and PD scores between the 1. and 2. trimester - MPO and MMP-8 did not increase until delivery - TIMP-1 amounts remained stable throughout the follow-up period. - Amounts of PMN elastase decreased continuously during the follow-up period	*Good*
Gürsoy et al. (2010b) [47]	Finland, prospective cohort study	Pregnant (30) Non-pregnant (24)	Pregnant: **T1:** Week 12–14 GA **T2:** Week 25–27 GA **T3:** Week 34–38 GA **T4:** 4–6 weeks postpartum **T5:** After lactation Non-pregnant: **T1-T3:** once per subsequent month	PPD, CAL, BOP, PI	*Saliva:* **Preparation:** not specified **Method:** Paraffin-stimulated saliva was collected by expectoration for 5 min **Processing:** salivary samples were diluted in assay buffer for 1 h **Storage:** Collected supernatants stored at −20 °C	Immunofluorometric assay, Gelatin zymography, ELISA	MMP8, MMP2, MMP9, MPO, TIMP1	**MMP-8 concentrations:** - Significantly lower than postpartum concentrations - Lowest during the second trimester and highest after delivery - Varying inversely to pregnancy gingivitis **MMP-2 and MMP-9 levels:** - highest level after lactation - Stable Elastase concentrations during follow-up - Significantly increased MPO concentrations after delivery. Controls: - Clinical and enzymological findings remained stable during follow-up period.	*Good*
Gürsoy et al. (2016) [50]	Finland, prospective cohort study	Pregnant (30) Non-pregnant (24)	Pregnant: **T1:** Week 12–14 GA **T2:** Week 25–27 GA **T3:** Week 34–38 GA **T4:** 4–6 weeks postpartum **T5:** After lactation Non-pregnant: **T1:** 1st month **T2:** 2nd month **T3:** 3rd month	BOP, PI	*Saliva:* **Preparation:***not specified* **Method:** Paraffin-stimulated saliva was collected by expectoration for 5 min **Processing:** *not specified* **Storage:** Collected supernatants stored at −70 °C	ELISA	17β-estradiol, Progesteron, Defensins (hBD-1, -2, -3, HNP-1)	- Reduced concentrations of hBD-1, hBD-2, and HNP-1 during pregnancy, especially during the third trimester - hBD-3 concentrations did not change - Weak associations between salivary defensins, hormone concentrations and clinical parameters.	*Good*
Öztürk et al. (2010) [48]	Turkey, longitudinal study	Pregnant (11)Non-pregnant (12)	**T1:** 1st trimester **T2:** 2nd trimester **T3:** 3rd trimester **T4:** 6–8 weeks postpartum	BOP, PI	*Saliva:* **Preparation:** Two hours prior to saliva collection, all subjects were instructed to eat a standard breakfast and to brush their teeth immediately afterwards **Method:** Passive drooling technique **Processing:** Centrifuged at 4 °C **Storage:** Collected supernatants stored at −24 °C	Ledwozyw’s method for LPO; Warren’s method for TSA; Lowry method for Total protein	LPO, TSA, Total protein level	**LPO levels:** - Significantly higher in all trimesters and postpartum - In the second trimester also significantly higher than in the third trimester and postpartum groups **TSA levels:** - Significantly higher in the second trimester group vs. non-pregnant, third trimester and postpartum groups - Significantly higher in the first trimester group vs. the postpartum group	*Fair*
Wu et al. (2016) [42]	China, Prospective case–control study	Pregnant (30) Non-pregnant (20)	Pregnant **T1:** Weeks 12–14 GA **T2:** Weeks 23–25 GA **T3:** Weeks 33–36 GA Non-pregnant **T1:** 1st month **T2:** 2nd month	PPD, CAL, BOP, PI, GBI	*GCF:* **Method:** Filter paper strips **Site:** Mesiobuccal sites of upper premolars **Collection time:** 30 s. **Processing:** Eluted in 200 μL phosphate-buffered saline and 2 μL of phenylmethanesulfonyl fluoride (20 mM) **Storage:** Stored at −70 °C	ELISA	IL-1β, TNF-α	- No significant changes in GCF TNF-𝛼 level in the pregnant group - GCF IL-1𝛽 level increased significantly during pregnancy - GBI and BOP increased significantly during pregnancy	*Good*
Yarkac et al. (2018) [43]	Turkey, case–control study with a periodontal therapy intervention	Pregnant (30) Non-pregnant (30)	**T1:** baseline **T2:** 3 weeks after periodontal treatment	PI, GI, PPD, CAL	*Saliva:* **Preparation:** Subjects were requested not to eat, brush, drink, or chew gum within 90 min of sample collection **Method:** Spitting into polypropylene tubes **Processing:** Centrifuged at 3220 rpm for 10 min **Storage:** Supernatants stored at −20 °C *GCF:* **Method:** Filter paper strips **Site:** Mesiobuccal sulcus of teeth in the anterior region **Collection time:** 30 s. **Processing:** not specified **Storage:** Stored at −20 °C	ELISA	IL-1β, IL-10	**Periodontal therapy:** - Major decrease in gingival inflammation in both groups - Decreased level of IL-1β in GCF in control group, but not in the test group - No difference in GCF IL-10 levels between the groups	*Strong*
Yarkac et al. (2021) [44]	Turkey, case–control study with a periodontal therapy intervention	Pregnant (30) Non-pregnant (30)	**T1:** baseline **T2:** 3 weeks after periodontal treatment	PPD, CAL, PI, GBI	*Saliva:* **Preparation:** Subjects were requested not to eat, brush, drink, or chew gum within 90 min **Method:** Spitting into polypropylene tubes **Processing:** Centrifuged at 3220 rpm for 10 min **Storage:** Collected supernatants stored at −20 °C *GCF:* **Method:** Periopaper strips **Site:** Mesiobuccal sulcus of randomly selected single-rooted upper anterior teeth with gingivitis **Collection time:** 30 s. **Processing:** Eluted in 200 µL phosphate-buffered saline **Storage:** Stored at −20 °C	ELISA	IL-6, IL-10	**Periodontal therapy:** - Pregnant women exhibited significantly deeper pockets and greater gingival inflammation after therapy - Levels of IL-6 in GCF are significantly higher in pregnant women after periodontal therapy - No differences in the levels of IL-10 observed	*Strong*

### 3.4. Differences in the Response of Inflammatory Biomarkers in Oral Biofilm between Pregnancy Stages

In total, six studies examined the differences in the response of inflammatory biomarkers in the oral biofilm throughout pregnancy stages. Periodontal parameters were reported to be significantly increased during early or late pregnancy stages compared to the postpartum period by two prospective studies [51,52]. While no differences in GCF cytokines levels (IL-1β, IL-1α, and TNF-α) were observed between pregnant and postpartum women [51], significantly lower salivary IL-1β and significantly higher salivary TNF-α levels were reported during late pregnancy stages compared to the postpartum period [52]. In a longitudinal study of 30 pregnant women from Finland, saliva samples were continuously taken each trimester, as well as twice after delivery, and analyzed for salivary estradiol, IL-1β, IL-8, MPO, MMP-8, MMP-9, MMP-2, and TIMP-1 levels [53]. The levels of MMP-2, MMP-9, TIMP-1, IL-1β, and IL-8 in saliva were steady during pregnancy, whereas salivary estradiol levels increased at each trimester of pregnancy and decreased significantly after delivery. MMP-8 significantly decreased between the first and second trimester and significantly increased after delivery. With regard to the association between salivary estradiol and inflammatory markers, statistically significant negative associations were found between estradiol and MPO concentration and MMP-8, while a positive association was detected between estradiol and TIMP-1. However, the authors did not record anthropometric parameters of the pregnant women in order to assess the correlations of salivary biomarker concentrations with factors such as weight history, diet or lifestyle during pregnancy.

Three studies tested the impact of periodontal interventions on periodontal variables as well as on GCF cytokine and PGE2 levels. After an 8-week intervention with intensive one-on-one oral hygiene counselling and non-surgical periodontal therapy among women of 16–24 weeks GA, periodontal variables decreased significantly, as did TNF-α and IL-1β levels [54]. Similarly, Yalcin and colleagues [55] reported significant improvements in all clinical parameters as well as a reduction in PGE2 levels throughout the trimesters after the implementation of a non-surgical periodontal therapy intervention plus oral hygiene instructions. In a randomized two-arm clinical trial with 67 pregnant women, Offenbacher and colleagues [56] found significantly reduced periodontal parameters and IL-1β levels among the intervention group receiving non-surgical periodontal therapy. The pregnant controls were associated with significant increases in PPD, PI, IL-1β and IL-1α. The strength of this intervention study is the recording and consideration of baseline criteria such as BMI, weight history, social status, education, smoking, etc. After adjustment, the authors presented the isolated effect of an oral intervention and considered the effects of pregnancy per se as a clinical inflammation parameter for peridontal stressors in isolation. Lifestyle and dietary behavior were not investigated.

**Table 3:** Differences in the response of inflammatory biomarkers in oral biofilm between pregnancy stages.

Author (year)	Country, Study Design	Investigated Groups (n of Subjects)	Measurement Interval	Periodontal Examination	Sample Collection	Sample Analysis	Evaluated Biomarker	Main study Findings and Key Messages	Quality Assessment
Bieri (2013) [51]	Switzerland, prospective consecutive case series	Pregnant (19)	**T1:** Week 12 GA **T2:** 4–6 weeks postpartum	PPD, CAL, BOP	*GCF:* **Method:** Endodontic paper points **Site:** Mesiobuccal aspects of all first molars **Collection time:** 15 s. **Processing:** Eluted in a lysis buffer and ethanol **Storage:** Stored at −79 °C	Quantitative rtPCR	IL-1α, IL-1β, IL-8, TNF-α	- BOP decreased from the 12th week of pregnancy to the 4–6 weeks postpartum - BOP scores and GCF levels of cytokines were not related to each other - No differences in GCF levels of cytokines between samples	*Fair*
Kaur et al. (2014) [54]	USA, clinical trial **Intervention:** Intensive one-on-one oral hygiene counselling and non-surgical periodontal therapy	Pregnant women with gingivitis (120)	**T1:** baseline **T2:** Week 4 GA (only PA examination) **T3:** Week 8 GA	PPD, CAL, BOP, PI, GI	*GCF:* **Method:** Filter paper strips **Site:** Gingival sulcus of two randomly selected sites on non-adjacent teeth **Collection time:** 30 s. **Processing:** Eluted in 100 μL phosphate-buffered saline **Storage:** Stored at −70 °C	ELISA	IL-1β, IL-6, TNF-α	**Periodontal intervention:** - Statistically significant reduction in all periodontal variables - Decreased levels of TNF-α and IL-1β	*Weak*
Gürsoy et al. (2014) [53]	Finland, longitudinal cohort study	Pregnant (30)	**T1:** Week 12–14 GA **T2:** Week 25–27 GA **T3:** Week 34–38 GA **T4:** 4–6 weeks postpartum **T5:** After lactation	PPD, CAL, BOP	*Saliva:* **Preparation:** not specified **Method:** Paraffin-stimulated saliva was collected by expectoration for 5 min **Processing:** Salivary samples were diluted in assay buffer for 1 h **Storage:** Supernatants stored at −20 °C	Immunofluorometric assay, Gelatin zymography, ELISA	IL-1β, IL-8, MMP8, MMP2, MMP9, MPO TIMP1, estradiol	- Estradiol concentrations associated positively with TIMP-1 and negatively with MPO and MMP-8 concentrations	*Fair*
Lasisi et al. (2018) [52]	Nigeria, longitudinal cohort study	Pregnant (47)	**T1:** 2nd or 3rd trimester **T2:** 3 months postpartum	PPD, CAL, BOP, PI	*Saliva:* **Preparation:** not specified **Method:** Spitting into a graduated universal bottle for a period of 10 min **Processing:** not specified **Storage:** Supernatants stored at −20 °C	ELISA	TNF-α, IFN-γ, IL-1β, Lactoferin, Lysozyme, β defensin-1	- Significantly lower levels of IL-1β and IFN-γ - Significantly higher levels of TNF-α - No significant difference in lactoferin, lysozyme and β defensin-1. - Level of IFN-γ correlated negatively with gingival index - Level of TNF-α correlated positively with gingival and periodontal indices - No significant difference in levels of salivary lactoferin, lysozyme and β defensin-1 in correlation to gingival and periodontal indices	*Fair*
Offen-bacher et al. (2006) [56]	USA, randomized two-arm clinical trial **Intervention:** Non-surgical periodontal therapy and oral health instructions on the use of a power toothbrush **Control:** Supragingival debridement and a manual toothbrush with no instructions	**Pregnant (67)** Intervention (35) Controls (32)	**T1:** baseline **T2:** 4–6 Weeks GA (intervention group) **T3:** 6 weeks postpartum (control group)	PPD, CAL, BOP, PI, GBI	*GCF:* **Method:** Filter paper strips **Site:** Mesio- and distobuccal sites from the two most posterior teeth in all four quadrants, excluding third molars **Collection time:** not specified **Processing:** Eluted in 100 μL phosphate-buffered saline **Storage:** Placed in liquid nitrogen	ELISA	IL-1β, IL-6, PGE2	**Periodontal intervention:** - Significant decreased incidence odds ratio for preterm delivery - Significant improvements in clinical status (CAL, PPD, PI, BOP, and GBI)	*Strong*
Yalcin et al. (2002) [55]	Turkey, intervention pilot study **Intervention:** non-surgical periodontal therapy	Pregnant women (22)	**T1:** 1st trimester **T2:** 2nd trimester **T3:** 3rd trimester	PPD, PI, GBI	*GCF:* **Method:** Filter paper strips **Site:** Mesiobuccal aspects of 2 teeth in the anterior region **Collection time:** 30 s. **Processing:** Eluted in tris buffered saline **Storage:** Stored at −70 ° C	ELISA	PGE2	**Periodontal intervention:** - Improvement in clinical parameters - Significant decrease in levels of PGE2 at the 2. and 3. trimesters	*Weak*

### 3.5. Differences in Inflammatory Responses in Oral Biofilm between Pregnant and Non-Pregnant Women with Periodontitis

We found two cross-sectional studies from Turkey that examined inflammatory responses in oral biofilm between pregnant women with gingivitis or periodontitis and non-pregnant women with gingivitis or periodontitis [57,58]. In the pregnant gingivitis and periodontitis groups, saliva TNF-α levels were shown to be significantly lower than in their control counterparts [57]. No such differences could be found for PGE2 levels. When comparing ANXA-1 and IL-1β levels, Hassan and colleagues [58] found that the pregnant gingivitis group exhibited nearly 2-fold higher ANXA-1 levels compared with the non-pregnant gingivitis group. No differences in IL-1β levels could be observed between the gingivitis or periodontitis group. However, among the pregnant subpopulations, women with gingivitis had significantly higher ANXA-1 and IL-1β levels than healthy pregnant women. Subpopulation differences were also observed for periodontal measurements. Within the non-pregnant or the pregnant subpopulations, the PPD and CAL in the periodontitis group were significantly higher than in the gingivitis groups. Whereas Gümüs and colleagues [57] excluded overweight patients with a BMI of 30 kg/m^2^ or more from their study, Hassan and colleagues [58] documented the age of participants without anthropometric data.

**Table 4:** Differences in inflammatory responses in oral biofilm between pregnant and non-pregnant women with periodontitis.

Author (year)	Country, Study Design	Investigated Groups (n of Subjects)	Measurement Interval	Periodontal Examination	Sample Collection	Sample Analysis	Evaluated Biomarker	Main study Findings and Key Messages	Quality Assessment
Gümüs et al. (2016) [57]	Turkey, cross-sectional study	**Pregnant women (59)** Healthy (17) Gingivitis (25) Periodontitis (17) **Non-pregnant women (70)** Healthy (27) Gingivitis (28) Periodontitis (15) **Postpartum women (47)** Healthy (14) Gingivitis (21) Periodontitis (12)	One time-point: 2nd or 3rd trimester for pregnant groups, 6 months postpartum for postpartum groups	PPD, CAL, BOP, PI	*Saliva:* **Preparation:** Subjects were asked to fast overnight during which individuals were requested not to drink (except water) or chew gum **Method:** Expectoration into polypropylene tubes for 5 min **Processing:** not specified **Storage:** Supernatants stored at −40 °C	ELISA	25-hydroxy-cholecalciferol (25(OH)D), PGE2, TNF-α	**Pregnant women vs. postpartum group:** - significantly lower TNF-α and PGE2 levels - significantly higher 25(OH)D3 levels **Control vs. postpartum group:** - Significantly higher TNF-α and 25(OH)D3 levels - Significantly lower PGE2 levels	*Fair*
Hassan et al. (2018) [58]	Turkey, cross-sectional study	**Pregnant women (78)** Healthy (22) Gingivitis (47) Periodontitis (9) **Non-pregnant women (69)** Healthy (30) Gingivitis (28) Periodontitis (11)	One time-point: 2nd or 3rd trimester	PPD, CAL, BOP, PI	*Saliva:* **Preparation:** Subjects were asked to fast overnight and to rinse their mouth with tap water 5 min prior to sample collection **Method:** not specified **Processing:** Centrifuged at 10,000× *g* for 15 min at 4 °C **Storage:** Supernatants stored at −80 °C	Protein assay kit, ELISA	Total protein, Annexin-1, IL-1𝛽	- Significantly higher levels of inflammatory markers in pregnant women with gingivitis compared with healthy pregnant and the respective non-pregnant group	*Good*

## 4. Discussion

In our review, we examined the currently available literature reporting on the response of inflammatory biomarkers in oral biofilm in the context of physiological changes during pregnancy. Our goal was to understand the relationship between these inflammatory responses and maternal, oral or systemic conditions. Beyond pregnancy, associations between periodontitis and other inflammatory comorbidities, such as diabetes mellitus (DM) Type 1 and 2, atherosclerosis, Alzheimer’s disease or inflammatory diseases like rheumatoid arthritis, have already been discussed in the literature [59]. However, risk profiles leading to an escalation of the inflammatory situation have not yet been clearly defined. Focusing on oral biofilm, our aim was to present the current state of research on inflammatory parameters in gingival fluid and/or saliva in relation to physiological changes in the context of pregnancy. In total, we included 39 studies in this review.

Overall, we found a high heterogeneity of studies in terms of study designs, methodology, and examined biomarkers. A fair number of studies showed an increased number of inflammatory biomarkers, such as elevated cytokine levels, among pregnant women compared to non-pregnant women [21,44,45,48]. Additionally, women with maternal or systemic conditions, e.g., with periodontitis, GDM or preeclampsia, showed significantly higher levels of inflammatory biomarkers in the oral biofilm compared to healthy pregnant or non-pregnant women [20,21,23,28,29,31,32,34,36,39]. It has been well established that due to hormonal changes and rapid weight gain during pregnancy, metabolic inflammation develops, activating inflammatory processes throughout the body [60]. However, while previous research has demonstrated this in pregnant women [61,62], the literature with a focus on periodontal health reveals inconclusive evidence. Hormonal changes during pregnancy can alter different pathways, including alterations of the microbiome, changing the integrity of periodontal ligament cells and finally modifying the immune response [63]. In total, information on the development of GDM as a possible cause of inflammatory processes in the oral cavity was provided in six studies. For example, Gümüs and colleagues [21] found a connection between the development of gingivitis and the development of GDM in the course of pregnancy. They showed that pro-inflammatory biomarkers are measurable in significantly higher concentrations in patients with gingivitis and proven GDM. They exclude obesity at the beginning of pregnancy as a possible additional trigger of silent inflammation. However, more detailed information on weight gain and nutritional behavior of the women during pregnancy was not provided. In this regard, examining whether diabetes is a consequence of the inflammatory process or responsible for it in some sense is essential. Future studies are warranted to obtain more conclusive findings of the association between inflammatory responses in oral biofilm and metabolic consequences during pregnancy and should, in our view, focus more on the mother as a whole.

In general, this raises the question of whether oral inflammatory biomarkers can be used to detect metabolic risks in mothers at an early stage of pregnancy in the future. As a consequence, analyses of the oral biofilm and determination of inflammatory mediators, as well as periodontal disease screening, might be used to assess the risk of pregnancy complications such as preterm birth, preterm LBW or PPROM [36,37,38,39]. However, due to a lack of standardized study conditions with sufficient sample sizes, no such analysis could be undertaken. Thus, no clear statement can be made at present.

Around 60% of the studies were cross-sectional, and therefore did not allow changes in inflammatory biomarkers to be studied over time. The few studies that did implement a longitudinal study design confirm that the intensities of inflammation vary depending on the status of pregnancy and in comparison to postpartum women [51,52,53]. The immunological dynamics and metabolic changes, from an anabolic state in early pregnancy to a catabolic state in late pregnancy [64], are accompanied by varying systemic cytokine patterns [8,65]. Although it is known that the extent of the change depends on both lifestyle and dental hygiene, none of the 39 studies, found under the given search string, investigated changes in the lifestyle of pregnant women in parallel with changes in the oral metabolome. Only five of the included studies were intervention studies, which investigated the influence of periodontal inflammation on the inflammatory process during pregnancy at the metabolome level. Previous studies have shown that periodontal therapy positively affects oral health by reducing periodontal parameters [66] and helps to reduce inflammatory and metabolic markers [67,68,69]. Furthermore, research suggests that non-surgical periodontal treatment could prevent pregnancy complications and consequently improve perinatal outcomes [70,71,72]. Similarly, based on our findings, non-surgical periodontal therapy interventions proved to be promising approaches for prevention and therapy of oral inflammation for the protection of mother and child.

As pointed out by Stadelmann and colleagues [73] in their systematic review on the association between GCF inflammatory mediators and adverse pregnancy outcomes, no generally applicable biomarker has been crystalized so far that would have the potential to be an inflammatory risk or pregnancy complication marker. Since it has not yet been possible to define specific inflammatory markers, including the preferred time-point for sample collection during pregnancy, the focus of included studies remains very heterogeneous, with biomarkers ranging from the inhomogeneous group of cytokines to periodontal parameters or enzymes and vasodilatation parameters such as PGE2.

The development of inflammatory processes and pregnancy complications is also decisively influenced by lifestyle, nutrition patterns, obesity and the age of the mother during pregnancy [74]. Diets with high intakes of processed meats, sugars, saturated fats, and high-fat dairy are especially associated with adverse pregnancy outcomes [75]. Furthermore, oral diseases are largely influenced by dietary behaviors, and studies demonstrate poor dietary habits to be an important risk factor for oral inflammation [76,77,78]. Both lifestyle and dental health are weighted differently for people depending on their culture, socioeconomic status and level of education [79]. Therefore, cultural and economic factors have been studied as affecting oral health outcomes [80] and were considered in the characterization of the women in several studies of this review. However, there were no effects on the results.

Although it is generally known that weight, weight gain, or central adiposity is influenced by diet and exercise and that obesity has an influence on inflammatory processes like periodontitis [81,82], only nine of the included studies recorded the BMI of their study participants [20,21,22,25,29,32,34,35,40]. However, BMI status was not further factored in for biomarker analysis. Solely two of the studies compared inflammatory responses in oral biofilm in relation to BMI category (normal weight vs. obese) [34,35]. In this respect, the study of Foratori and colleagues [34] confirms the relationship between inflammatory processes and weight. Previous research demonstrates that excessive gestational weight gain is known to be associated with multiple adverse fetal and maternal outcomes [83]. Unfortunately, weight trajectory over the course of pregnancy and the postpartum period is not investigated in the included studies.

Another important factor to consider is nutritional pattern, which plays an essential role for both maternal and oral health. In a previous cross-sectional study, we identified a significant association between higher adherence to the DASH (Dietary Approaches to Stop Hypertension) and Mediterranean diets and lower odds to be affected by periodontal diseases [82]. The reduction of oxidative stress and the increased intake of antioxidants through certain nutritional components seem to have beneficial effects on gingival and periodontal inflammation due to the modified host immune response. In addition, Bartha and colleagues [84] found that, following a 6-week Mediterranean diet intervention, higher diet adherence showed significant negative correlations with all assessed oral inflammatory parameters. Thus, it seems these nutritional patterns are protective against periodontitis. In our review, only two studies recorded information on dietary patterns among their study subjects [25,40]. Studying oxidative stress markers in saliva, Zygula and colleagues [25] found lower salivary MDA levels among women with GDM that were treated with a dietary intervention compared to insulin-treated subjects. Hence, future studies should consider the influence of longtime lifestyle habits, including oral healthcare, exercise, stress assessment, as well as the nutritional and dietary patterns of pregnant women (before and during pregnancy) when investigating the activity of inflammatory processes in the oral biofilm. Therefore, pregnant women’s health care might be served inter-professionally, including the input of dentists, gynecologists, midwives and nutritionists.

### Limitations

We identified several limitations that should be cautiously considered when interpreting the results of this review. Due to the largely cross-sectional nature of the included studies, one cannot define the directionality between the inflammatory processes, periodontal health and systemic conditions. The included studies utilized inconsistent and heterogeneous approaches in grouping their data. Various methodologies for analyzing biomarkers found in saliva or GCF, as well as types of inflammatory biomarkers, were reported. As a consequence, this complicates the comparison of findings across studies. Coming to a common consensus and finding particular biomarker patterns was not possible. Standardized methods for sample collection and biomarker analysis are needed to ensure comparable results while enhancing study reproducibility. Additionally, due to the lack of study subject’s data on other possible determinants, e.g., nutrition patterns, anthropometric data, demographics, socioeconomic status, etc., associations with oral inflammation might be under- or overestimated.

## 5. Conclusions

Changes at the hormonal, immunological and metabolic level influence the microbiota of the mother during pregnancy in the intestine, vagina and oral cavity. The health status before the beginning of pregnancy plays an essential role. A healthy diet- and lifestyle influences the development of diseases or pregnancy complications and can counteract inflammation at various levels. Multiple approaches to the analysis of inflammatory biomarkers suggest specific interactions also in the oral cavity but show heterogeneous results and thus confirm the complexity of the processes. Since the oral cavity may become a site of bacterial entry due to gingival inflammation or periodontitis, further studies focusing on the interplay between maternal, oral and metabolic health in the context of pregnancy are essential. Biomarkers of the oral microbiome can, in our view, become valuable markers of inflammation when considered in the context of diet and lifestyle, as well as the present metabolic and hormonal situation of the expectant mothers.

## Data Availability

Not applicable.

## Supplementary Materials

The following supporting information can be downloaded at: https://www.mdpi.com/article/10.3390/nu14224894/s1, Table S1: Final search strategy.
