# Supplementary material for: Inflammatory Response in Oral Biofilm during Pregnancy: A Systematic Review
Source: Nutrients. 2022 Nov 19;14(22):4894. doi: 10.3390/nu14224894 (PMC9694722; doi:10.3390/nu14224894)
Supplement: Supplementary file 1 [file nutrients-14-04894-s001.zip › nutrients-1909061-supplementary.pdf]

Table S1: Overview of biomarkers and their function

Biomarker (abbreviation)	Function
**25-hydroxy-cholecalciferol (25(OH)D)**	25-Hydroxyvitamin D3<br>Modulation of periodontal inflammation
**8-hydroxy-2′-deoxyguanosine (8-OHdG)**	Biomarker for oxidative stress and DNA-damage
**A proliferation-inducing ligand (APRIL)**	Member of TNF-superfamily<br>Immune system development and signaling
**Aldehyde dehydrogenase (ALDH)**	Enzymes for oxidation of aldehydes<br>Protects oral cavity from aldehydes
**Annexin-1**	Inhibits innate immune cells and promotes T-cell activation
**B-cell activating factor (BAFF)**	Member of TNF-superfamily<br>Proliferation and differentiation of B cells
**C-reactive protein (CRP)**	Acute phase protein<br>Activation of complement system
**Cystatins**	Protease inhibitors<br>Regulate tissue-destructive protease activities in the oral cavity Regulate complement activation<br>Protective effect under inflammatory conditions
**Defensins (human beta defensins (hBD-1, -2, -3); human neutrophil peptides (HNP)-1)**	Antimicrobial peptides<br>Form pores and trigger lysis in bacteria<br>Immune signaling, chemotactic to T-cells
**Extracellular vesicle-CD63+**	Surface marker CD63+ containing extracellular vesicles<br>Intercellular communication<br>Proinflammatory
**Glutathion peroxidase (GPx)**	Antioxidant enzyme protecting tissues against oxidative damage
**Interleukin (IL)-1α**	Proinflammatory cytokine
**IL-1β**	Proinflammatory cytokine<br>Stimulator of prostaglandin synthesis<br>Role in physiologic process of parturition
**IL-6**	Proinflammatory cytokine<br>Stimulation of acute phase protein and PGE2 synthesis
**IL-6sR**	Proinflammatory cytokine
**IL-8**	Proinflammatory chemokine<br>Angiogenesis<br>Cleavage of MMPs<br>Tissue damage
**IL-10**	Anti-inflammatory cytokine

	Inhibition of proinflammatory cytokines
**IL-17**	Proinflammatory cytokine<br>Increases TNF-α activity<br>Stimulator of prostaglandin synthesis
**IL-33**	Cytokine<br>Induces helper T-cells, mast cells, eosinophils
**INF-γ**	Proinflammatory cytokine<br>Innate and adaptive immunity<br>Activates macrophages
**Lactoferrin**	Antimicrobial protein
**Leptin**	Signaling molecule<br>Modulates proliferation, protein synthesis
**Lysozyme**	Antimicrobial protein
**Malondialdehyde (MDA)**	Lipid peroxidation product, biomarker for oxidative stress
**Matrix-metalloproteinase 2 (MMP-2)**	Gelatinase<br>Degradation of extracellular matrix, tissue remodeling
**MMP-8**	Collagenase<br>Degradation of extracellular matrix, tissue remodeling
**MMP-9**	Gelatinase<br>Degradation of extracellular matrix, tissue remodeling
**Monocyte chemoattractant protein-1 (MCP-1)**	Proinflammatory cytokine
**Myeloperoxidase (MPO)**	Lysosomal antimicrobial enzyme secreted by neutrophils in inflammation<br>Formation of Neutrophil Extracellular Traps (NETs)
**N-acetyl-β-D-hexosaminidase (HEX)**	Salivary exoglycosidase<br>Degradation of oligosaccharide chains in organogenesis, growth and normal tissue turnover
**Neutrohil elastase (NE)**	Antimicrobial protease secreted by neutrophils in inflammation<br>Formation of Neutrophil Extracellular Traps (NETs)
**Osteoprotegerin (OPG)**	Member of TNF-superfamily<br>Cytokine receptor<br>Immune system development and signaling
**Placental alkaline phosphatase (PLAP)**	Allosteric enzyme<br>Indicator for placenta-derived exosomes
**Superoxide dismutase**	Antioxidant enzyme protecting tissues against oxidative damage
**Placental growth factor (PlGF)**	Member of VEGF-family, angiogenic<br>Biomarker for preeclampsia

**Polymorphonuclear neutrophil (PMN)-elastase**	Antimicrobial protease<br>Degrades host extracellular matrix components and to causes Tissue breakdown
**Prostaglandin E2 (PGE2)**	Inflammatory mediator<br>Role in physiologic process of parturition
**Soluble fms-like tyrosine kinase 1 (sFlt-1)**	Binds VEGF and PlGF, anti-angiogenic<br>Biomarker for preeclampsia
**Soluble intercellular adhesion molecule-1 (sICAM)**	Proinflammatory marker<br>Transmigration of leukocytes
**Soluble receptor activator of NFκB ligand (RANKL)**	Member of TNF-superfamily<br>Immune mediator<br>Cell proliferation and apoptosis
**Superoxide dismutase**	Antioxidant enzyme protecting tissues against oxidative damage
**Thiobarbituric acid-reactive substances (TBARS)**	Biomarker for lipid peroxidation<br>Measure of reactive oxygen species damage during inflammation
**Tissue inhibitor of MMP-1 (TIMP-1)**	Inhibition of MMP-1<br>Transportation and stabilization of MMPs
**Tumor necrosis factor alpha (TNF-α)**	Proinflammatory cytokine<br>Activation of immune cells
**TNF-R1**	TNF-Receptor
**TNF-R2**	TNF-Receptor
**Uric acid**	Antioxidant parameter
**Vascular endothelial cell growth factor (VEGF)**	Signaling protein<br>Angiogenesis<br>Integrity of oral mucosa
**β-galactosidase (GAL)**	Salivary exoglycosidase<br>Degradation of oligosaccharide chains in organogenesis, growth and normal tissue turnover
**α-mannosidase (MAN)**	Salivary exoglycosidase<br>Degradation of oligosaccharide chains in organogenesis, growth and normal tissue turnover
**α-frucosidase (FUC)**	Salivary exoglycosidase<br>Degradation of oligosaccharide chains in organogenesis, growth and normal tissue turnover
**β-glucuronidase (GLU)**	Salivary exoglycosidase<br>Degradation of oligosaccharide chains in organogenesis, growth and normal tissue turnover
